# Carotid Intima-Media Thickness and Plasma Asymmetric Dimethylarginine in Mexican Children Exposed to Inorganic Arsenic

**DOI:** 10.1289/ehp.1205994

**Published:** 2013-06-11

**Authors:** Citlalli Osorio-Yáñez, Julio C. Ayllon-Vergara, Guadalupe Aguilar-Madrid, Laura Arreola-Mendoza, Erika Hernández-Castellanos, Angel Barrera-Hernández, Andrea De Vizcaya-Ruiz, Luz M. Del Razo

**Affiliations:** 1 Departamento de Toxicología, Centro de Investigación y de Estudios Avanzados del IPN, México D.F., México; 2 Hospital Español, México D.F., México; 3 Unidad de Investigación y Salud en el Trabajo, Instituto Mexicano del Seguro Social, México D.F., México; 4 Departamento de Biociencias e Ingenieria CIIEMAD-IPN, México D.F., México

## Abstract

Background: Arsenic exposure is a risk factor for atherosclerosis in adults, but there is little information on arsenic and early risk biomarkers for atherosclerosis in children. Carotid intima-media thickness (cIMT) is an indicator of subclinical atherosclerotic burden that has been associated with plasma asymmetric dimethylarginine (ADMA), a predictor of cardiovascular disease risk.

Objectives: The aim of this study was to investigate associations of arsenic exposure with cIMT, ADMA, and endothelial adhesion molecules [soluble intercellular cell adhesion molecule-1 (sICAM-1); soluble vascular cell adhesion molecule-1 (sVCAM-1)] in children who had been exposed to environmental inorganic arsenic (iAs).

Methods: We conducted a cross-sectional study in 199 children 3–14 years of age who were residents of Zimapan, México. We evaluated cIMT using ultrasonography, and plasma lipid profiles by standard methods. We analyzed ADMA, sICAM-1, and sVCAM-1 by ELISA, and measured the concentrations of total speciated arsenic (tAs) in urine using hydride generation cryotrapping atomic absorption spectrometry.

Results: In the multiple linear regression model for cIMT, tAs categories were positively associated with cIMT increase. The estimated cIMT diameter was greater in 35- to 70-ng/mL and > 70-ng/mL groups (0.035 mm and 0.058 mm per 1-ng/mL increase in urinary tAs, respectively), compared with the < 35-ng/mL group. In addition to tAs level, plasma ADMA was a significant predictor of cIMT. In the adjusted regression model, cIMT, percent iAs, and plasma sVCAM-1 were significant predictors of ADMA levels (e.g., 0.419-μmol/L increase in ADMA per 1-mm increase in cIMT).

Conclusions: Arsenic exposure and plasma ADMA levels were positively associated with cIMT in a population of Mexican children with environmental arsenic exposure through drinking water.

Citation: Osorio-Yáñez C, Ayllon-Vergara JC, Aguilar-Madrid G, Arreola-Mendoza L, Hernández-Castellanos E, Barrera-Hernández A, De Vizcaya-Ruíz A, Del Razo LM. 2013. Carotid intima-media thickness and plasma asymmetric dimethylarginine in Mexican children exposed to inorganic arsenic. Environ Health Perspect 121:1090–1096; http://dx.doi.org/10.1289/ehp.1205994

## Introduction

Inorganic arsenic (iAs) is naturally occurring and ubiquitous in the environment. In most populations, drinking water is the main source of human exposure. Long-term exposure to iAs has been associated with coronary disease, stroke, ischemic heart disease, hypertension, and carotid atherosclerosis in adults (States et al. 2009). Epidemiologic studies conducted in Taiwan have demonstrated that long-term iAs exposure is significantly associated with carotid atherosclerosis in adults, with a positive dose–response relationship after adjustment for other cardiovascular risk factors, which suggests that iAs exposure may be an independent risk factor for atherosclerosis (Wang et al. 2002, 2007). However, to our knowledge, there have been no epidemiologic studies on the role of iAs exposure in atherosclerosis initiation or progression in pediatric populations. Atherosclerosis is a multistage disease that can initiate in childhood and remain subclinical until adulthood, when it becomes clinically manifest. Carotid artery wall intima-media thickness (cIMT) is a widely accepted indicator of subclinical atherosclerotic burden, and its determination could be useful in identifying young adults at risk for premature coronary atherosclerosis (Slyper 2004). Epidemiologic evidence suggests a close association between cIMT and plasma asymmetric dimethylarginine (ADMA) concentrations (Ayer et al. 2009). ADMA at baseline predicted subsequent cardiovascular disease in a 22-year follow-up study of adult women (Leong et al. 2008). Clinical conditions with elevated plasma ADMA concentrations in children included hypertension, hypercholesterolemia, chronic kidney disease, and diabetes mellitus (Tain and Huang 2011). However, there is little toxicologic evidence regarding ADMA. The association of ADMA with adverse clinical events could be related to the attenuation of the vascular protective effects of nitric oxide (NO). In animal models, local inhibition of NO accelerates early neointima formation (Cayatte et al. 1994). Moreover, Nanayakkara et al. (2005) found a positive association between ADMA and soluble vascular adhesion molecule-1 (sVCAM-1), a molecule expressed in activated endothelial cells, in patients with mild to moderate renal failure.

The aim of the present study was to investigate the association between iAs exposure and cIMT, plasma ADMA, and endothelial adhesion molecules in a pediatric population exposed to environmental iAs.

## Materials and Methods

*Study participants*. A cross-sectional study was conducted in 199 children (3–14 years of age) who were residents of the Zimapan region in Mexico. This study was approved by the Institutional Review Board of CINVESTAV-IPN (Centro de Investigación y de Estudios Avanzados del Instituto Politécnico Nacional). In this area, high concentrations of naturally occurring iAs are frequently found in the bedrock and consequently in underground and surface waters (Armienta et al. 1997). The children were recruited from two local schools and were residents of five area towns (Calvario, Llano Norte, Aguacatal, Muhi, and Downtown). At the time of evaluation, arsenic levels measured in the drinking water of these towns ranged from 3 to 135 ng As/mL. Before enrollment in the study, the parents read and signed a written informed consent form. Parents were interviewed by trained interviewers on general characteristics, with an emphasis on the source of drinking water, marine food consumption, secondhand smoking exposure, detailed residential information including whether the mother lived in Zimapan area during pregnancy (yes/no), child allergies, child surgery interventions, medication, and child medical history. Only children with a minimum of 1 year of residency in the Zimapan region were eligible to participate. Children with diabetes or cardiovascular disease were excluded.

*Child examination and sample collection.* Children were examined by an expert cardiologist who was blinded to the study design and participants’ clinical data. The children were examined using a cardiovascular ultrasound system (Vivid i®; General Electric, Milwaukee, WI, USA) equipped with a 14-MHz linear transducer; the examiner followed a standardized protocol using B-mode ultrasound with the child in a supine position with the head turned slightly to the left and then right (Pignoli et al. 1986). cIMT was calculated based on automatic contour detection of the intima and media layers in a user-defined search region along the vessel wall. Multiple cIMT measurements were made between pairs of intima and adventitia points along the posterior wall of the vessel. The following parameters were calculated: average cIMT (cIMTmean), maximum cIMT (cIMTmax), and minimum cIMT (cIMTmin).

We measured body weight and height using standard protocols. We calculated the body mass index (BMI) using the formula: weight (kilograms)/height (meters squared). The BMI *z*-score was calculated, with BMI categorized based on guidelines of the Centers for Disease Control and Prevention (2000). Each participant provided a first morning void urine sample. Urinalysis was performed immediately, and samples were stored at –20°C at the local health clinic until they were transported with cooling blocks to Mexico City for further analysis. A sample of approximately 12-hr fasting venous blood was collected. Plasma was prepared from blood samples by centrifugation at 4°C and stored at –80°C.

*Analysis of As in water and urine.* Arsenic analysis included the analysis of sodium arsenite (NaAs^III^O_2_) and arsenic acid disodium salt (Na_2_HAs^V^O_4_). Both of these chemicals (> 99% pure) and dimethylarsinic acid [DMAs^V^; as (CH_3_)_2_As^V^O(OH); 99% pure] were obtained from Sigma Chemical Co. (St. Louis, MO, USA). Methylarsonic acid (MAs^V^) disodium salt [CH_3_As^V^O(ONa)_2_; 99% pure] was obtained from Ventron (Danvers, MA, USA). Working standards of these arsenicals, which contained 1 µg As/mL, were prepared daily from stock solutions. Sodium borohydride (NaBH_4_) and l-cysteine hydrochloride were obtained from EM Science (Gibbstown, NJ, USA). Ultrapure Tris-hydrochloride monohydrate was purchased from Sigma, and Tris-hydrochloride was purchased from J.T. Baker (Phillipsburg, NJ, USA). All other chemicals used were at least analytical grade.

The concentrations of total arsenic (tAs) in drinking water were determined by HG (hydride generation)–atomic fluorescence spectrometry, as previously described (Le and Ma 1998). Trace elements in standard water reference material [SRM 1643e; National Institute of Standards and Technology (NIST), Gaithersburg, MD, USA] containing 60.4 ± 0.7 ng As/mL were used for quality control. HG-atomic absorption spectrometry with a cryotrap for the capture and separation of hydrides was used for the analysis of iAs and its metabolites in urine (Hernández-Zavala et al. 2008). We used standard reference material (SRM 2669) from NIST for quality control in measurements of arsenic species in frozen human urine. We used SRM 2669 level 1 and level 2 to validate the analysis of arsenic species at low and elevated concentrations in the urine matrix, respectively. The low tAs concentration urine sample at SRM 2669 level 1 had a reference value of 9.22 ± 0.32 ng As/mL, and the reference value was 43.67 ± 0.63 ng As/mL for the high tAs concentration at SRM 2669 level 2. Replicate analyses of SRM 2669 showed values with < 10% coefficient of variation of reference values for the high and low standards. The sum of the concentrations of iAs, MAs, and DMAs in urine was reported as tAs.

*Plasma analyses*. The concentrations of glucose, total cholesterol, triglycerides, and high-density lipoprotein (HDL) were measured in plasma by the end-point enzymatic method using fully automatic biochemistry analyzer (SYNCHRON LX 20; Beckman Coulter, Mexico). Values of very low-density lipoprotein (VLDL) and low-density lipoprotein (LDL) were calculated using the Friedewald formula (Friedewald et al. 1972). The atherogenic index was calculated for each child as total cholesterol/HDL. The hematocrit, hemoglobin, and leucocytes were determined using a standard method. A second plasma aliquot was stored at –70°C and analyzed after one thaw cycle for ADMA using an enzyme-linked immunosorbent assay (ADMA human ELISA Kit; Immundiagnostik AG, Lörrach, Germany). ADMA was assessed in duplicate (intra-assay coefficient of variation < 15%), and the average was recorded as the ADMA level. Two controls were included in the kit assay to control for quality. The mean (range) were 0.25 (0.19–0.32) µmol/L for control level 1 and 0.76 (0.57–0.95) µmol/L for level 2. In each assay, the values of the controls were in the range established by the manufacturer. In addition to ADMA quantification, adhesion molecules, such as sVCAM-1 and soluble intercellular adhesion molecule-1 (sICAM-1) were analyzed using ELISA (Invitrogen, Carlsbad, California, USA).

*Evaluation of iAs exposure and metabolism*. The concentration of tAs in the urine was used to estimate individual exposure to iAs. The proportion of arsenic in each species (%iAs, %MAs, and %DMAs) was calculated by dividing the concentration of arsenic in each species by the tAs in urine.

**Figure 1:**
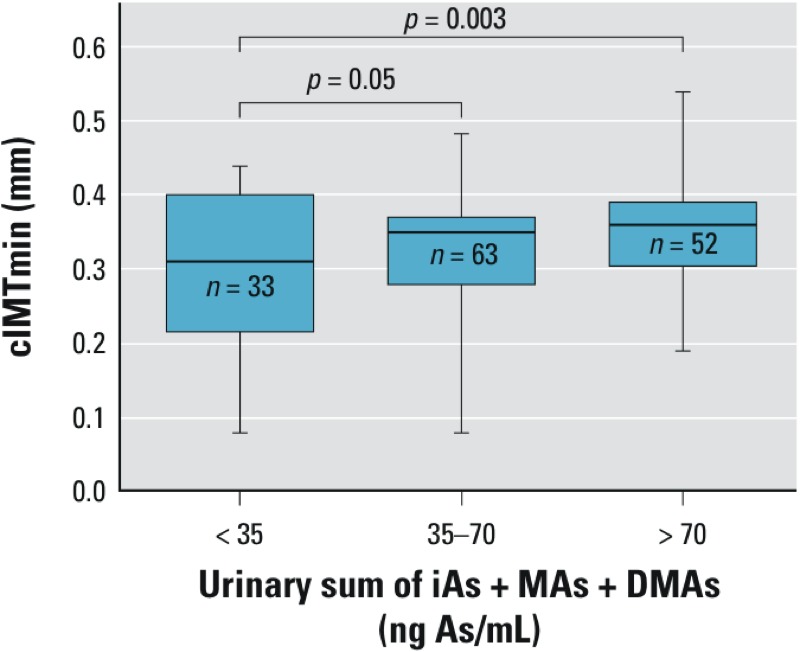
Box plots for cIMT by level of sum of inorganic and methylated arsenic species in urine of children. The outer bounds of the boxes represent the interquartile range; the median is represented by the midline. The whiskers represent minimum and maximum values. Analysis of variance was performed to assess the cIMT increase over tAs categories. Additionally, Wilcoxon-type test was used to evaluate trends across urinary arsenic categories (*p* = 0.027).

*Statistical analyses*. We performed exploratory analyses to assess data quality and consistency and the distribution of the variables of interest. Continuous variables presented non-normal distribution except for ADMA and %MAs, which were normally distributed. All continuous variables are described as geometric mean and range, and also the mean ± SD are reported. Frequencies or percentages are reported for categorical variables. We used simple linear regression models to estimate associations of ADMA and cIMT, with potential confounders (age, sex, BMI or *z*-score, atherogenic index, lipid serum profile, adhesion molecules, and fasting plasma glucose) and with urine tAs and arsenic species. We also evaluated the effect of *in utero* exposure by simple linear regression analyses on the two outcomes of interest. In addition to evaluating urine tAs as a continuous variable, we stratified exposure into three categories: < 35 ng/mL [where 35 ng/mL represents the Biological Exposure Index (BEI) or permissible limit for occupational As exposure (American Conference of Governmental Industrial Hygienists 2004)], 35–70 ng/mL, and > 70 ng/mL (twice the BEI value). Additionally, a Wilcoxon-type test for trend was performed to evaluate cIMT increase across arsenic categories (Figure 1). Pearson’s correlation coefficient (*r*_P_) for plasma ADMA associations and Spearman’s correlation coefficient (*r*_S_) for plasma sVCAM-1 and sICAM-1 were performed among the main exposure–outcome association. Multivariable linear regression analyses of associations with ADMA or cIMT were adjusted for potential confounding variables related to outcome, based on Wald tests with a *p*-value of < 0.20, or if their inclusion improved the model fit (based on the change in 10% of *R*^2^ value). The adjusted models also included age, which did not fulfill this statistical criterion but was considered to be biologically important. In the case of cIMT, the explanatory variable in the linear regression model was modeled as an untransformed continuous variable or as square transformed continuous variable. Because inferences based on square transformed cIMT were comparable (data not shown), results are reported for cIMT as an untransformed variable for easier interpretation. Analysis for validation of the multiple regression with robust weight function were performed for cIMTmin analyses (Davidson and McKinnon 1993). Validation of the multiple regression ADMA model was performed using studentized residuals. Model predictions were graphed against standardized residuals to assess heteroscedasticity (Montgomery and Peck 1992). *p*-Values < 0.05 were considered statistically significant. All statistical analyses were performed using STATA version 10 (StataCorp, College Station, TX, USA).

## Results

*Characteristics of the study population*. Of the initial 199 child participants, three had no urine or blood samples, one was excluded due to an atrial septal defect, and 47 children had no ultrasonography. Most of the children (70%) were < 6 years old (age range, 3–14 years) (Table 1). In total, 79% of children had urinary tAs values higher than the BEI of 35 ng/mL, and only 21% showed levels below this reference value. Using standard international BMI age- and sex-specific cut-off points, 66% were classified as normal weight, 7% underweight, 18% overweight, and 9% obese (Table 1). There were no significant differences between boys and girls in the parameters studied (data not shown), except for glucose (86.11 mg/dL in boys vs. 80.03 mg/dL in girls; *p* = 0.0001), HDL (53.85 vs. 50.7 mg/dL; *p* = 0.0327), hemoglobin (13.76 vs. 14.00%; *p* = 0.025), and sICAM-1 (0.59 vs. 0.64 µg/mL; *p* = 0.0142).

**Table 1:** Child characteristics, urinary arsenicals levels, plasmatic biomarkers, and cIMT of study population in Zimapan, Mexico.

Variable	*n*	Percent or GM (range)	Mean ± SD
Sex
Male	107	54
Female	92	46
*In utero* exposure
Yes	164	82
No	35	18
Age (years)	199	5.1 (3–14)	5.26 ± 1.49
≤ 5	140	70
> 5	59	30
BMI (kg/m^2^)	195	16.02 (11.8–26)	16.17 ± 2.31
BMI *z*-score (percentile)	195	40.01 (1–99)	55.27 ± 30.68
Urinary arsenic (ng/mL)	195
iAs		5.41 (0.57–100.78)	8.70 ± 11.61
MAs		5.41 (0.21–55.67)	7.98 ± 8.31
DMAs		46.69 (4.93–236.95)	57.27 ± 40.22
tAs		59.14 (5.71–369.94)	74.31 ± 57.04
Urinary arsenic (%)	195
iAs		9.15 (2.33–72.08)	10.27 ± 6.21
MAs		9.14 (1.50–17.98)	9.67 ± 3.07
DMAs		78.95 (24.77–91.52)	79.59 ± 8.51
Plasma analysis	193
Glucose (mg/dL)		83 (61–130)	83 ± 11
Total cholesterol (mg/dL)		147 (13–284)	150 ± 29
Triglycerides (mg/dL)		73 (20–333)	81 ± 42
HDL cholesterol (mg/dL)		51 (30–103)	52 ± 12
LDL cholesterol (mg/dL)		79 (20–168)	82 ± 22
VLDL cholesterol (mg/dL)		15 (4–67)	16 ± 8
Atherogenic index		2.86 (1.2–5.3)	2.92 ± 0.58
Hemoglobin (g/dL)	194	13.86 (11.7–17.1)	13.89 ± 0.87
Hematocrit (%)	194	39.78 (33.5–48.7)	39.84 ± 2.32
Leucocytes (no./mL)	194	6.35 (3.4–12.8)	6.52 ± 1.56
Cardiovascular risk biomarkers
ADMA (μmol/L)	196	0.69 (0.23–1.43)	0.72 ± 0.19
sVCAM-1 (μg/mL)	177	1.50 (0.68–4.56)	1.56 ± 0.50
sICAM-1 (μg/mL)	196	0.60 (0.33–1.42)	0.62 ± 0.15
Carotid ultrasonography (mm)^*a*^	152
cIMTmin		0.32 (0.08–0.54)	0.33 ± 0.08
cIMTmean		0.43 (0.24–0.8)	0.44 ± 0.07
cIMTmax		0.54 (0.28–1.28)	0.55 ± 0.13
GM, geometric mean. ^***a***^cIMT values are minimum, mean, and maximum.			

Only 22% of the population reported any seafood consumption, and only 16% were exposed to secondhand smoke. Neither seafood consumption nor secondhand smoke were significant predictors of cIMT or ADMA (data not shown).

In unadjusted models, triglycerides, VLDL, sICAM-1, and sVCAM-1 levels were significant predictors of plasma ADMA concentration, and age was a marginally significant predictor (*p* = 0.06) (Table 2). The association among plasma ADMA and urinary tAs, MAs, and DMAs concentration had a *p*-value < 0.1. Sex, BMI *z*-score, and BMI categories did not predict plasma ADMA. Atherogenic index and BMI *z*-score were significant predictors of cIMTmin, and overweight and sICAM-1 were marginal predictors, whereas age and sex were not associated with cIMT (Table 2). In addition, cIMTmin diameter was positively associated with both unstratified and stratified urinary tAs (with a monotonic increase in geometric mean cIMT with increasing categorical exposure). Compared with the lowest exposure group, estimated values of cIMT were 0.033 mm and 0.054 mm higher among those with tAs 35–70 and > 70ng/mL, respectively (Table 2).

**Table 2:** Relation of children characteristics, lipid serum profile, plasma adhesion molecules, and urinary arsenic exposure with plasma ADMA or cIMTmin.

Variable	ADMA (μmol/L)		cIMTmin (mm)	
	β (95% CI)	*p*-Value	β (95% CI)	*p*-Value
Age (years)	–0.017 (–0.034, 0.0007)	0.061	0.005 (–0.005, 0.016)	0.290
Sex	0.041 (–0.012, 0.093)	0.130	0.010 (–0.016, 0.036)	0.451
BMI (kg/m^2^)	0.0055 (–0.006, 0.017)	0.353	0.006 (0.0004, 0.012)	0.037
BMI *z*-score (percentile)	0.0006 (–0.0002, 0.002)	0.147	0.0006 (0.0002, 0.0010)	0.005
BMI categories
Underweight	0.011 (–0.096, 0.119)	0.836	–0.023 (–0.073, 0.026)	0.348
Overweight	0.049 (–0.021, 0.12)	0.168	0.029 (–0.005, 0.063)	0.090
Obesity	0.029 (–0.064, 0.12)	0.54	0.011 (–0.035, 0.057)	0.633
Plasma analyses
Triglycerides (mg/dL)	0.0007 (0.00002, 0.0013)	0.043	0.0002 (–0.000082, 0.0005)	0.146
VLDL cholesterol (mg/dL)	0.0034 (0.00007, 0.007)	0.046	0.0012 (–0.00041, 0.0027)	0.147
Atherogenic index	0.0047 (–0.041, 0.051)	0.84	0.024 (0.0017, 0.046)	0.035
Adhesion molecules
sICAM-1 (μg/mL)	0.00024 (0.000065, 0.0004)	0.007	0.00008 (–5.16e–6, 0.00017)	0.065
sVCAM-1 (μg/mL)	0.0001 (0.000045, 0.00015)	0.000	0.00002 (–3.55e–6, 4.7e–5)	0.092
tAs categories (ng/mL)
35–70	–0.017 (–0.09, 0.055)	0.640	0.033 (–0.0004, 0.067)	0.053
> 70	0.010 (–0.064, 0.085)	0.785	0.054 (0.019, 0.089)	0.003
Urinary As (ng/mL)
tAs	0.0004 (–0.00007, 0.0009)	0.092	0.0002 (7.48e–6, 0.0005)	0.043
iAs	0.0012 (–0.0011, 0.004)	0.306	0.0009 (–0.0002, 0.002)	0.124
MAs	0.0028 (–0.0005, 0.006)	0.093	0.0013 (–0.0003, 0.003)	0.101
DMAs	0.0006 (–0.00006, 0.0013)	0.076	0.0003 (0.00003, 0.0007)	0.034
Males were compared with females. BMI categories were compared with normal weight category. tAs categories were compared with < 35 ng/mL category. Simple linear regression analyses were used to compare untransformed ADMA or cIMTmin with continuous variables or categorical data.				

*Association of cIMT with iAs exposure*. cIMTmin was significantly associated with urine tAs based on simple linear regression (Figure 1) and after adjustment for atherogenic factor, BMI *z*-score, age, and plasma ADMA (Table 3). In contrast, tAs exposure was not correlated with plasma lipids or BMI (*p* > 0.05; data not shown). The multivariable regression model explained 18% of the variability in cIMTmin diameter, with the strongest associations estimated for plasma ADMA concentration (0.068-mm increase; 95% CI: 0.0117, 0.124 for a 1-µmol/L increase in ADMA) and tAs > 70 ng/mL (0.058-mm increase; 95% CI: 0.0198, 0.095 compared with tAs < 35 ng/mL) (Table 3).

**Table 3:** Robust multivariable linear regression analysis of associations between cIMTmin and arsenic levels and cardiovascular markers in children.

Explanatory variable	β^*a*^ (95% CI)	*p*-Value
tAs in urine (35–70 ng/mL)^*b*^	0.035 (–0.0028, 0.072)	0.070
tAs in urine (> 70 ng/mL)^*b*^	0.058 (0.0198, 0.095)	0.003
Plasma ADMA (μmol/L)	0.068 (0.0117, 0.124)	0.018
Atherogenic index	0.019 (–0.0007, 0.038)	0.059
BMI* z*-score (percentile)	0.0005 (0.00007, 0.0009)	0.023
Age (years)	0.008 (–0.0011, 0.016)	0.088
*R*^2^ = 0.18; *p* = 0.0000; *n* = 141. ^***a***^Average difference in cIMT (mm) per unit change in the explanatory variable. ^***b***^Sum of inorganic and methylated ­arsenic species.		

*Associations of cardiovascular biomarkers with iAs exposure and metabolism*. Plasma ADMA, sICAM-1, and sVCAM-1 were highly correlated (*p* < 0.05), and ADMA and sICAM biomarkers were also correlated with triglycerides and VLDL (see Supplemental Material, Table S1). BMI, glucose, total cholesterol, HDL, LDL, and the atherogenic index were not significantly correlated with cardiovascular biomarkers (data not shown). ADMA was weakly correlated with urinary tAs (*r*_P_ = 0.122; *p* = 0.092), MAs (*r*_P_ = 0.121; *p* = 0.093), and DMAs (*r*_P_ = 0.128; *p* = 0.076). sVCAM-1 was significantly correlated with age (*r*_S_ = –0.17; *p* = 0.024).

**Figure 2:**
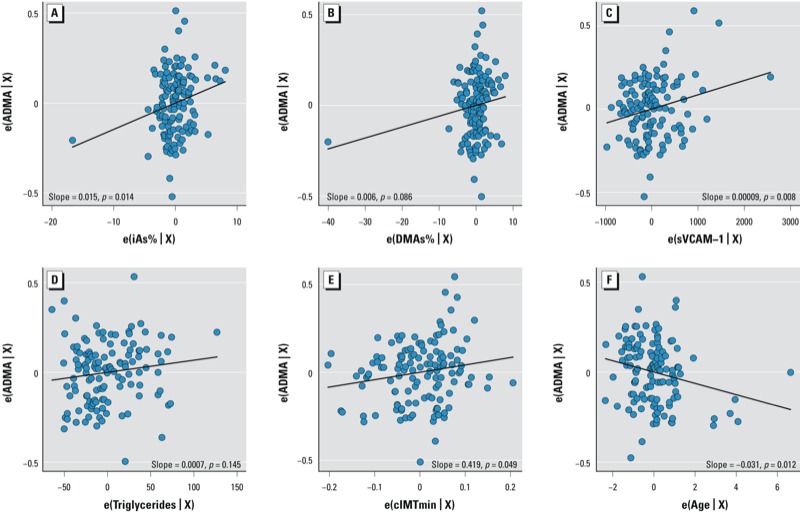
Predictors of plasma ADMA (µmol/L) in children according to multivariable linear regression. (*A*) Urinary %iAs, (*B*) urinary %DMAs, (*C*) plasma sVCAM-1, (*D*) plasma triglycerides, (*E*) cIMTmin, and (*F*) age, where e(...X) contains the regressor X after controlling for its relationship with other regressors.

In multivariable regression analysis, %iAs, sVCAM-1, and cIMTmin were significantly associated with plasma ADMA (Table 4, Figure 2). The model explained 15% of the variability in plasma ADMA, with the strongest predictors being cIMTmin diameter (0.419-µmol/L increase in ADMA per 1-mm cIMTmin), age (0.0314-µmol/L decrease per year), and %iAs (0.0147-µmol/L increase per 1-unit %iAs). Age was a stronger predictor of ADMA than was %iAs. %DMAs and triglycerides were also significant predictors of ADMA. Finally, values of ADMA or cIMT diameter were not significantly associated with *in utero* arsenic exposure (*p* > 0.05; data not shown).

**Table 4:** Multivariable linear regression analysis of associations between plasma ADMA (µmol/L) in children and explanatory variables.

Explanatory variable	β^*a*^ (95% CI)	*p*-Value
iAs (%)	0.0147 (0.003, 0.026)	0.014
DMAs (%)	0.006 (–0.0009, 0.0129)	0.086
sVCAM-1 (μg/mL)	0.000086 (0.00002, 0.00015)	0.008
Triglycerides (mg/dL)	0.00067 (–0.0002, 0.0016)	0.145
cIMTmin (mm)	0.4189 (0.0010, 0.837)	0.049
Age (years)	–0.0314 (–0.056, –0.0069)	0.012
*R*^2^ = 0.19; adjusted *R*^2 ^= 0.151; *p* = 0.0002; *n* = 128. ^***a***^Average difference in ADMA in μmol/L per unit change in the explanatory variable.		

## Discussion

Over recent decades, exposure to iAs in central Mexico and potential effects on human health have given rise to much concern (Del Razo et al. 2011). In our study of a pediatric population, we found that cIMT and plasma ADMA were increased in association with As levels in urine.

Previous studies have reported that cIMT is increased in children at high risk for cardiovascular disease because of familial hypercholesterolemia, type 1 diabetes, having parents with premature myocardial infarction, or elevated levels of ultrasensitive C-reactive protein compared with other children (Jarvisalo et al. 2001; Slyper 2004). Epidemiologic studies have also reported associations between long-term iAs exposure and outcomes related to atherosclerosis in adults, including ischemic heart disease, cerebrovascular disease, and peripheral vascular disease (Simeonova and Luster 2004). To our knowledge, ours is the first epidemiologic study to report an association between iAs exposure and cIMT in children. Specifically, we estimated a significant association between urinary tAs (35–70 ng/mL and > 70 ng/mL) and cIMTmin in children, 70% of whom were < 6 years old. In the present study, cIMTmin was significantly associated with ADMA and urinary tAs. This suggests that the minimum cIMT diameter increase could represent the earliest morphologic changes related to arsenic exposure and/or ADMA increase.

Few studies have focused on early cardiovascular effects of iAs exposure in children. One study based on autopsy findings for five children from an As-endemic area of Chile reported intimal thickening in the small- and medium-sized arteries; this similar vascular lesion was found in heart, stomach, intestines, and mesentery (Rosenberg 1974). In other autopsy studies, the earliest abnormalities observed in children 2–15 years of age with atherosclerotic risk factors were fatty streaks and fibrous plaques (Berenson et al. 1998; Newman et al. 1991). Endothelial dysfunction may be the initial phenomenon in subclinical atherosclerosis that precedes thickening in the vascular wall (Kallio et al. 2010). An increase in cIMT could be a consequence of effects of iAs on endothelial dysfunction, foam cell formation stimulation, reactive oxidative stress production, proinflammatory chemokines and cytokines, inflammation, vascular smooth muscle cell proliferation, endothelial cell proliferation, platelet aggregation, and decreased fibrinolytic activity (Balakumar and Kaur 2009; Simeonova and Luster 2004; Wang et al. 2002).

In multivariable regression analyses, urine tAs, plasma ADMA, and the atherogenic index were significant predictors of increased cIMT. But lipid serum profile was not correlated with tAs in our pediatric study population (data not shown), as shown in one report that confirms our results and is based on adults in Taiwan with exposures to high levels of arsenic and ischemic heart disease (Hsueh et al. 1998). Although lipids in serum are intimately related to atherosclerosis, our results suggest that early iAs-mediated effects on atherogenesis may be independent of the lipid serum profile. Median concentrations of plasma total cholesterol, HDL, VLDL, LDL, and triglycerides were consistent with levels recommended for the primary prevention of atherosclerosis beginning in childhood (Kavey et al. 2003) and were within the normal range reported previously for Mexican children (Perichart-Perera et al. 2007).

Although we did not find an association between *in utero* arsenic exposure and cIMT diameter or ADMA level, we cannot rule out a contribution of *in utero* arsenic exposure to the association between urine arsenic in childhood and cIMT because most of the mothers lived in the area during pregnancy (83%). Srivastava et al. (2007) hypothesized that accelerated development of aortic lesions and vasorelaxation defects observed in Apo E^–/–^ mice exposed to arsenic *in utero* was attributable to an arsenic-mediated reduction in NO availability. Our findings suggest that the association between urinary tAs and cIMTmin in our pediatric study population could have been mediated by an effect of iAs on ADMA, which is an endogenous inhibitor of NO.

Adhesion molecules and ADMA have been studied in children at risk of cardiovascular disease due to hypertension, obesity, and a family history of cardiovascular disease (Ayer et al. 2009; Goonasekera et al. 2000). Adhesion molecules, such as sICAM-1 and sVCAM-1, have been used as early biomarkers of atherosclerosis because of their participation in the initial step of the disease, in which they promote the translocation of monocytes and leucocytes to the arterial endothelium with subsequent migration to the subendothelial space, initiating the atherosclerosis process (Chen et al.2007; Glowinska et al. 2003). Although sICAM-1 may be a less specific marker than sVCAM-1, which is primarily expressed by activated endothelial cells and muscle cells in atherosclerotic plaques (Blake and Ridker 2002), it is more predictive of cardiovascular disease in apparently healthy subjects (de Lemos et al. 2000). sVCAM-1 and sICAM-1 have been correlated with iAs exposure in adults (Chen et al. 2007). In contrast, we did not find significant correlations between both adhesion molecules and any arsenicals in the urine. Prospective cohort studies have reported that the plasma concentration of sICAM-1 is elevated many years before an initial myocardial infarction (Hwang et al. 1997; Ridker et al. 1998) but sICAM-1 has also been reported to be elevated in children with acute otitis media caused by bacterial infections (Liu et al. 2010). In contrast with the adhesion molecules, ADMA was associated with cIMT and with the relative proportion of iAs in our study population. In adults with peripheral arterial occlusive disease, there is a progressive reduction in urinary nitrate and cGMP rates (markers of NO formation), which may be caused partly by accumulation of ADMA (Böger et al. 1997). ADMA plasma concentrations in patients with end-stage renal disease were higher in hemodialysis patients with manifested atherosclerosis disease compared with hemodialysis patients without atherosclerosis disease (Kielstein et al. 1999). Nevertheless, additional research is needed to establish the utility of ADMA as a biomarker of environmentally mediated cardiovascular disease.

Urine %MAs was associated with carotid atherosclerosis in a previous case–control study of adults (Wu et al. 2006). %MAs was not a significant predictor of cIMT in our study population, but %DMAs was weakly associated with plasma ADMA concentration in the multivariable model (*p* = 0.086). This result could be explained by differences in methylation capacity between children and adults. Children in Bangladesh were reported to have lower urinary %MAs and higher %DMAs than adults, suggesting that the second step in arsenic methylation may be more active in children (Chowdhury et al. 2003). We found a positive and significant association between urine %iAs and plasma ADMA in the multivariable regression analysis (Table 4). A recent study of surgical samples from three coronary heart disease patients who lived in an arsenic area in Chile reported that iAs was the predominant arsenic species in cardiovascular tissue, whereas DMAs and MAs levels in the same samples were relatively low or undetectable (e.g., iAs concentration in the auricle was 49.2 µg/g, whereas MAs and DMAs were undetectable (Roman et al. 2011). In Apo E^–/–^ mice, an animal model of atherosclerosis, atheroma formation in arsenic-exposed mice was accompanied by increasing levels of iAs in the vessel wall (Simeonova et al. 2003).

The relative proportion of iAs in urine was significantly associated with plasma ADMA in our pediatric study population, in whom many potential confounding factors, such as smoking, diabetes, and sedentary lifestyle, were absent. This suggests that ADMA might affect the arterial wall early in life, rather than being a biomarker of age-related vascular degeneration only, and that ADMA might play a role in early iAs-mediated atherosclerotic effects. ADMA is an endogenous inhibitor of nitric oxide synthase (NOS) that is derived from the proteolysis of proteins containing methylated arginine residues. NO has been characterized as the “endogenous antiatherosclerotic molecule” due to its antithrombotic, antioxidant, and vasodilatation properties, among others (Böger 2003). Therefore, any condition that reduces NO may promote atherosclerosis. A study of adults in Inner Mongolia, China, exposed to high levels of arsenic in well water reported a negative association between iAs exposure and stable plasma metabolites of NO, including nitrite and nitrate (NO_x_) (Pi et al. 2000). Interestingly, the most robust correlation with NO_x_ depletion was %iAs in blood (*p* < 0.001). Although we did not measure iAs in blood, multivariable regression analysis showed that %iAs in the urine was associated with plasma ADMA. Although several mechanisms underlying iAs exposure–mediated NO depletion have been proposed, the association is not completely understood (Kumagai and Pi 2004). We believe that our findings suggest a new pathway in which iAs exposure could decrease NO levels and promote atherosclerotic disease. However, evidence of NO-independent effects of ADMA on microvascular lesions in NOS-knockout and wild-type mice has also been reported (Suda et al. 2004).

To our knowledge, ours is the first epidemiologic study to implicate ADMA in subclinical atherosclerosis due to iAs exposure. In an experimental study of myelin alteration in rats exposed to iAs via drinking water (36 µg/mL), plasma ADMA was significantly increased 4 months after treatment compared with the control group [9.7 ± 0.6 vs. 3.4 ± 0.6 nmol/mL (Zarazua et al. 2010)].

We cannot rule out the possibility of bias due to confounding by factors such as a family history of stroke, cardiovascular disease, or diabetes, and the temporal relations between exposure and the outcomes that we evaluated cannot be established due to the cross-sectional nature of our analysis. However, additional studies are warranted given our findings of an association of iAs with cIMT and plasma ADMA in children.

## Supplemental Material

(156 KB) PDF
