# Supplementary material for: Carotid Intima-Media Thickness and Plasma Asymmetric Dimethylarginine in Mexican Children Exposed to Inorganic Arsenic
Source: Environ Health Perspect. 2013 Jun 11;121(9):1090–6. doi: 10.1289/ehp.1205994 (PMC3764073; doi:10.1289/ehp.1205994)
Supplement: (156 KB) PDF [file ehp.1205994.s001.pdf]

Supplemental Material

Carotid Intima-Media Thickness and Plasma Asymmetric Dimethylarginine in Mexican Children Exposed to Inorganic Arsenic

Citlalli Osorio-Yáñez, Julio C. Ayllon-Vergara, Guadalupe Aguilar-Madrid;Laura Arreola-Mendoza, Erika Hernández-Castellanos, Angel Barrera-Hernández, Andrea De Vizcaya-Ruiz, and Luz M. Del Razo

Supplemental Material, Table S1. Pearson or Spearman correlation coefficients and the p value of association between plasma ADMA, sVCAM-1 and sICAM concentration, urinary arsenicals, plasma biomarkers and cIMT in Mexican children exposed to iAs.

Variable	N	ADMA (μmol/L)		sVCAM-1 (ng/mL)		sICAM-1(ng/mL)	
		r_p	p	r_s	p	r_s	p
Age (year)	195	-0.134	0.061	-0.169	0.024	-0.09	0.218
BMI (kg/m^2)	195	0.067	0.353	0.015	0.85	0.086	0.235
iAs (ng/mL)	195	0.074	0.306	0.017	0.825	0.099	0.174
MAs (ng/mL)	195	0.121	0.093	0.063	0.407	0.044	0.544
DMAs (ng/mL)	195	0.128	0.076	-0.0003	0.997	0.029	0.692
tAs (ng/mL)	195	0.122	0.092	-0.004	0.959	0.055	0.449
Tryglicerides (mg/dL)	195	0.146	0.044	0.064	0.405	0.169	0.019
VLDL Cholesterol (mg/dL)	195	0.144	0.046	0.067	0.378	0.169	0.019
ADMA (μmol/L)	195			0.198	0.008	0.176	0.013
sVCAM-1 (ng/mL)	177	0.264	0.0004			0.282	0.0001
sICAM-1 (ng/mL)	195	0.191	0.0072	0.282	0.0001		
cIMTmin (mm)	152	0.196	0.016	0.107	0.217	0.132	0.108
cIMTmean (mm)	152	0.006	0.938	0.124	0.155	0.081	0.321
cIMTmax (mm)	152	-0.108	0.187	0.082	0.349	0.065	0.431
